# Flexural Strength of Acrylic Resin Denture Bases Processed by Two Different Methods

**DOI:** 10.5681/joddd.2014.027

**Published:** 2014-09-17

**Authors:** Jafar Gharechahi, Nafiseh Asadzadeh, Foad Shahabian, Maryam Gharechahi

**Affiliations:** ^1^Professor of Prosthodontics, Dental Research Center, School of Dentistry, Mashhad University of Medical Sciences, Mashhad, Iran; ^2^Associate Professor of Prosthodontics, Dental Research Center, School of Dentistry, Mashhad University of Medical Sciences, Mashhad, Iran; ^3^Assistant Professor of Prosthodontics, Dental Research Center, School of Dentistry, Ahvaz University of Medical Sciences, Ahvaz, Iran; ^4^Assistant Professor of Endodontics, Dental Research Center, School of Dentistry, Mashhad University of Medical Sciences, Mashhad, Iran

**Keywords:** Acrylic resin, denture base, physical processes, strength

## Abstract

***Background and aims.*** The aim of this study was to compare flexural strength of specimens processed by conventional and injection-molding techniques.

*** Materials and methods.*** Conventional pressure-packed PMMA was used for conventional pressure-packed and injection-molded PMMA was used for injection-molding techniques. After processing, 15 specimens were stored in distilled water at room temperature until measured. Three-point flexural strength test was carried out. Statistical analysis was carried out by SPSS using t-test. Statistical significance was defined at P<0.05.

***Results.*** Flexural strength of injection-polymerized acrylic resin specimens was higher than that of the conventional method (P=0.006). This difference was statistically significant (P=0.006).

***Conclusion.*** Within the limitations of this study, flexural strength of acrylic resin specimens was influenced by the molding technique.

## Introduction

Acrylic resin polymers have been introduced as denture base materials and the majority of denture bases are fabricated using polymethylmethacrylate (PMMA). These materials have optimal physical properties and excellent esthetics with relatively low toxicity compared to other plastic denture bases.^1^ Compression molding with heat activation in a water bath for resin polymerization is the conventional method to process dentures.^2^ However, shrinkage and dimensional change of denture bases during resin polymerization is unavoidable and has been well documented.^1^ Mechanical behavior of the denture base, including flexural strength, depends on the type of the material and even on processing techniques.^3^ Therefore, acrylic resins and processing methods have been modified to improve physical and chemical properties of denture bases. One example is the introduction of injection-molding technique.

In 1942, Pryor^4^ introduced the injection-molding technique to overcome the adverse effects of compression molding. Grunewald et al^5^ investigated Pryor’s technique and reported no significant advantages over the conventional method. Several injection-molded denture base materials and processing techniques are now available, with each claimed to produce denture bases with better properties.^6,7^ Ivocolar acrylic resins are one of the important resins among complete denture materials.^1^ Therefore, in this study, Ivocolar acrylic resins were used as a control (SR-Ivocap Triplex Hot) and experimental groups (SR-Ivocap High Impact), which had the most similarities in chemical structures according to manufactures’ claims. Previous studies have compared injection and conventional molding methods by use of acrylic resins with different systems and brands with principal differences in chemical structures.^8-10^ As a result, in the current study we tried to use the materials with the most similarity. Other drawbacks and shortcomings of previous studies were the fact that they evaluated flexural strength by production of specimens with various shapes (dentures and denture-shaped specimens). In the present study, rectangular specimens were examined and thus, variables such as shape, size and thickness of the samples were controlled.

The aim of this study was to compare the flexural strength of rectangular resin specimens cured by conventional processing method versus SR-Ivocap injection-molding system.

## Materials and Methods

In this study, flexural strength of conventional pressure-packed PMMA (SR-Ivocap Triplex Hot, Ivoclar Vivadent, Liechtenstein) was compared to those of injection-molded PMMA base material (SR-Ivocap High Impact, Ivoclar Vivadent, Liechtenstein).

**Figure 1.**
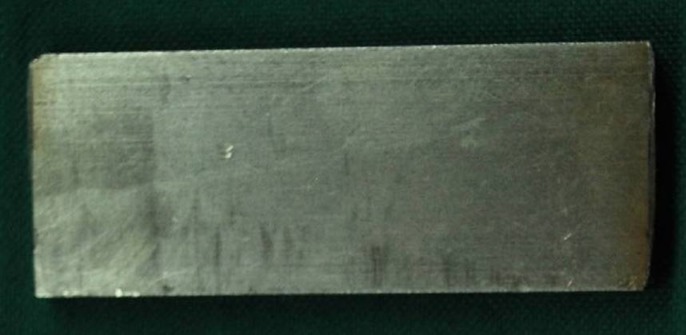
Rectangular stainless steel plates.

**Figure 2.**
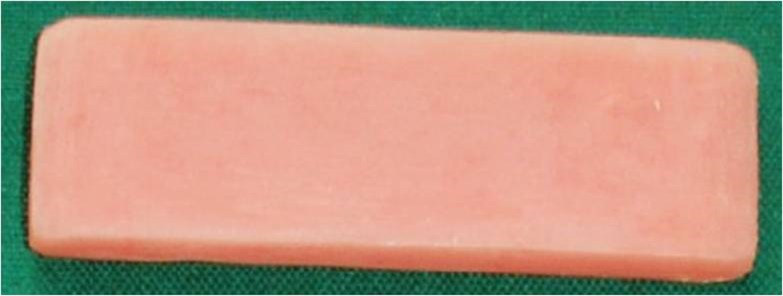
Acrylic resin specimens.

Three rectangular stainless steel plates were fabricated (Figure 1) to prepare 15 acrylic resin specimens (Figure 2) with a nominal size of 50×20×4 mm, for conventional and injection-molding methods.

Two layers of wax (Cavex, Netherlands) were placed on stainless steel plates and flasked. To embed the flasks, type III dental stone (Herodent; Vigodent, Petropolis, RJ, Brazil) was used and mixed according to manufacturer's instructions (100 g of powder with 30 mL of water).

The liquid-to-powder ratio of SR Triplex Hot resin was 10 mL of liquid to 23 g of powder. The acrylic resin was mixed according to manufacturer’s recommendations and packed in the flask. Conventional PMMA specimens were fabricated using a conventional flasking and pressure-pack technique. Polymerization of the resin was carried out in boiling water under a pressure of 100 N for 45 minutes. After polymerization, the curing flasks were bench-cooled to room temperature, and the specimens were deflasked. The surfaces were finished using 800-, 400- and 200-grit sandpapers (Norton; Saint-Gobain Abrasivos, Brasil).

For the injection-molded technique, the specimens were flasked according to manufacturer’s instructions using the Ivocap flask. Premeasured SR-Ivocap capsules of resin and monomer (20 g powder, 30 mL monomer) were mixed in Cap vibrator (Ivoclar AG) for 5 minutes before injecting into the flask. For curing process of the SR-Ivocap system, hydraulic pressure of 6 atm at 100°C was maintained for 35 minutes. A 10-minute cooling process using running water with a pressure of 6 atm was used before deﬂasking the denture. Finally, there was a further l0-minute cooling period, but without any extra pressure. Then the specimens were dedeflasked and the surfaces were finished using 800-, 400- and 200-grit sandpapers (Norton; Saint-Gobain Abrasivos, Brasil).

**Figure 3.**
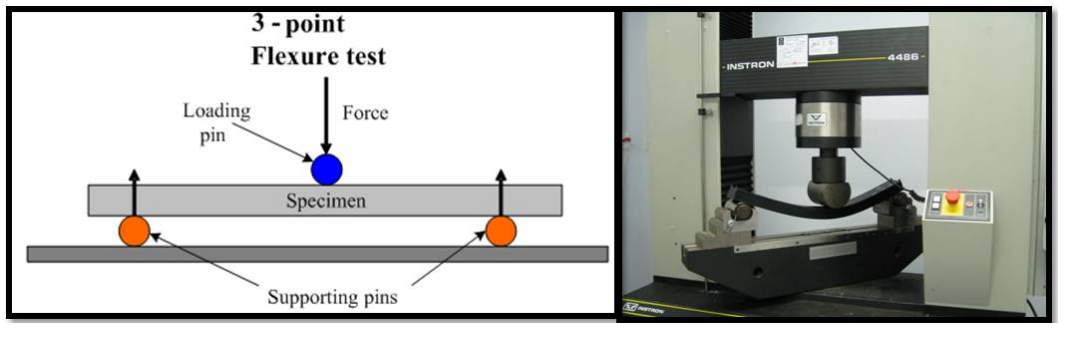
Universal testing machine for Three-point flexural strength test.

All the specimens were stored in distilled water at room temperature for 10 days before flexural strength test. The tests were carried out immediately after retrieving the specimens from distilled water without drying the specimens. For flexural test, a universal testing machine (Model STM-50, Santam, Tehran, Iran) was used (Figure 3). The distance between the specimen supports was 40 mm and the loading force was applied to the specimens at a crosshead speed of 5 mm/min until the specimens fractured. The diameter of loading and supporting plunger was 20 mm.

The maximum load exerted on the specimens was recorded, and the flexural strength was calculated according to the following formula:^11^

F=3WL/2bd^2^

[F: flexural strength; W: load at fracture; L: distance between supporting points (40 mm); b: width of specimens (mm); d: specimen thickness (mm)]

Flexural strength was calculated in MPa.

### Statistical Analysis

Following data collection, statistical analysis was carried out by SPSS (SPSS Inc., Chicago, IL, USA) using t-test. Statistical significance was defined at P<0.05.

## Results

A summary of the flexural strength in conventional pressure-packed and injection-molded SR-Ivocap is shown in Table 1. Flexural strength of injection-polymerized acrylic resin specimens was higher than that of the conventional method. T-test showed that the difference was statistically significant (P=0.006).

**Table 1:** Means and standard deviations of flexural strength of acrylic specimens

Molding method	X±SD	n	Min	Max
Conventional molding	107.99±12.89429	15	86.08	125.87
Injection molding	122.475±7.16493	15	112.27	130.76

## Discussion

This study evaluated the flexural strength of rectangular specimens produced using injection-molding and compression-molding techniques with thermally activated PMMA resin. Conventional method is the most applicable method for curing acrylic resin due to its simplicity and relatively good accuracy and in various studies this method has been considered the gold standard for comparison with the other techniques.^2^ Among denture processing methods, injection molding has always been interesting for researchers because of compensation of polymerization shrinkage due to the pressure exerted by injection of the acrylic resin.^1^

Wolfaardt^12^ reported that many different factors affected physical properties of acrylic resin dentures. Factors such as size and shape,^13^ denture thickness,^14^ different types of denture base materials,^15^ and presence of teeth^16^ can influence the physical and mechanical characteristics of bases during denture processing. Many studies have evaluated the physical properties of denture bases by production of different specimens with various shapes. Therefore, it is better to use specimens with simple shapes for comparison of properties instead of dentures and denture-shaped specimens. In the present study, rectangular specimens were examined and thus, variables such as shape, size, and thickness of the samples were controlled. By this approach, the physical properties were directly related to acrylic resin itself. Similar to the present study, Salim^17^ and Baydas^18^ used rectangular acrylic resin plates for their evaluation. In contrast, complete denture bases were utilized in separate research studies by Jackson,^19^ Nogueira,^20^ Abby^21^ and Venus.^22^

In most studies, only the molding technique has been considered as variables that affect the physical and mechanical properties of dentures and less attention has been focused on the effect of different types of acrylic resins used for molding. Differences in acrylic resin brands may be considered another variable in addition to molding technique, affecting the mechanical properties. In such studies, in addition to the method of molding, the type of the resin was also different in each group. Therefore, the results could not merely be attributed to the type of the molding process itself.^23-26^As a result, in the present study, denture base resins with the same composition were used (produced by Ivocolar Vivadent/Liechtenstein), which were processed by two different techniques. Therefore, comparisons were made only between the molding techniques and the effect of material type was eliminated.

The denture base may fracture due to different reasons such as improper fitting, anatomical notches, and lack of adequate design. The fracture takes place due to flexure fatigue when the denture base is loaded and the maximum mechanical capacity of the material is exceeded.^27^ The flexural strength is one of most important mechanical properties of resin materials and it has been reported that acrylic resins with incomplete polymerization have lower mechanical properties compared to those with complete polymerization.^28-30^Thus, by measuring the flexural strength, the quality of polymerization might be evaluated to some extent in addition to determination of denture base resistance to force and trauma.^29^

Three-point flexural test, adopted by international standards for polymer materials, including ISO 1567:1999 Dentistry-Denture base polymers, is the most common technique of measuring flexural properties of denture bases.^31^ In this study, a loading force was applied to specimens at a crosshead speed of 5 mm/min based on a study by Barbosa.^32^ According to ISO 1565, flexural strength of acrylic resin, processed and cured with any method, should be no less than 65 MPa.^33^ The results of this study demonstrated that the mean flexural strength of the two curing methods tested in the current work was higher than that required by ISO 1565. Thus, both molding techniques are suitable for clinical use. However, the flexural strength of SR-Ivocap injection method was significantly higher than that of conventional pressure-packed curing, exhibiting higher resistance and stability of denture bases fabricated by the injection method.

Ganzarolli et al^34^ demonstrated higher flexural strength for the injection-molding technique compared to the conventional method. Hamanaka^35^ reported that all the injection-molded thermoplastic resins had significantly higher impact strengths compared to the conventional PMMA. Uzun^33^ compared the fracture resistance of six acrylic resin denture base materials through impact and transverse strength tests. Three rapid heat-polymerized resins, two high-impact strength resins and a strengthened injection-molded acrylic resin (SR-Ivocap) were included in the study. Among these acrylic resins, SR-Ivocap resin showed the highest impact strength values. Ucar^36^ reported lower flexural strength for SR-Ivocap injection-molding technique than that in the present study (69.8 MPa versus 122 MPa). This might be accounted for by the fact that specimens tested in their study were kept at room-temperature distilled water for 100 days while specimens in the present study were stored in water for 10 days. Increased water sorption in 100 days might have decreased the flexural strength.

In heat-cured acrylic resins, the amount of residual monomer and also the mechanical properties have a close relationship with the polymerization condition.^28-30^ Harrison reported that a wide range of curing cycles without terminal boiling resulted in residual monomer 3 times more than that when terminal boiling was performed.^30^ Residual monomer can influence flexural strength of denture bases due to its plasticizing properties.^29,32^ Given this concept and the high flexural strength of SR Ivocap specimens in this study, it may be concluded that in these specimens, the amount of residual monomer was less than that in the conventional processing technique and the polymerization was more complete. Also, the good physical and mechanical properties of the injection-molded resin might be attributed to dual polymerization and small particle sizes.^34^

Thus, according to the results of previous and present studies, it would seem that the injection-molding method has the advantages of resistance and stability over the conventional molding technique.

## Conclusion

Flexural strength of acrylic resin specimen was influenced by molding technique and SR-Ivocap injection procedure had higher flexural strength compared to conventional molding.

## References

[1] Takamata T, Setcos JC (1989). Resin denture bases: review of accuracy and methods of polymerization. Int J Prosthodont.

[2] Zarb GA, Bolender CL, Carlsson GE (1997). Boucher’s prosthodontic treatment for edentulous patients, 11th ed.

[3] Lee HH, Lee CJ, Asaoka K (2012). Correlation in the mechanical properties of acrylic denture base resins. Dent Mater J.

[4] Pryor WJ (1942). Injection molding of plastics for dentures. J Am Dent Assoc.

[5] Grunewald AH, Paffenbarger GC, Dickson G (1952). The effect of molding processes on some properties of denture resins. J Am Dent Assoc.

[6] Garfunkel E (1983). Evaluation of dimensional changes in complete dentures processed by injection-pressing and the pack-and-press technique. J Prosthet Dent.

[7] Anderson GC, Schulte JK, Arnold TG (1988). Dimensional stability of injection and conventional processing denture base acrylic resin. J Prosthet Dent.

[8] Trage R (1980). Experience gained with the SR-lvocap system. Quintessence Int.

[9] Schmidt KH (1975). The SR-Ivocap system and the structure of denture bases. Quintessenz.

[10] Jackson AD, Grisius RJ, Fenster RK, Lang BR (1989). Dimensional accuracy of two denture base processing methods. Int J Prosthodont.

[11] Yunus N, Rashid AA, Azmi LL, Abu-Hassan MI (2005). Some flexural properties of a nylon denture base polymer. J Oral Rehabil.

[12] Wolfaardt J, Cleaton-Jones P, Fatti P (1986). The influence of processing variables on dimensional changes of heat-cured poly (methyl methacrylate). J Prosthet Dent.

[13] Barco MT Jr, Moore BK, Swartz ML, Boone ME, Dykema RW, Phillips RW (1979). The effect of relining on the accuracy and stability of maxillary complete dentures--an in vitro and in vivo study. J Prosthet Dent.

[14] Woelfel JB, Paffenbarger GC, Sweeney WT (1960). Dimensional changes occurring in dentures during processing. J Am Dent Assoc.

[15] Woelfel J8, Paffenbjrger GC, Sweeney WT (1965). Clinical evaluation of complete dentures made of eleven different types of denture base materials. J Am Dent Assoc.

[16] Da Breo EL, and Herman P (1991). A new method of measuring dimensional change. J prosthet Dent.

[17] Salim S, Sadamori S, Hamada T (1992). The dimensional accuracy of rectangular acrylic resin specimens cured by three denture base processing methods. J Prosthet Dent.

[18] Baydas S, Bayindir F, Akyil MS (2003). Effect of processing variables (different compression packing processes and investment material types) and time on the dimensional accuracy of polymethyl methacrylate denture bases. Dent Mater J.

[19] Jackson AD, Grisius RJ, Fenster RK, Lang BR (1989). Dimensional accuracy of two denture base processing methods. Int J Prosthodont.

[20] Nogueira SS, Ogle RE, Davis EL (1999). Comparison of accuracy between compression- and injection-molded complete dentures. J Prosthet Dent.

[21] Abby A, Kumar R, Shibu J, Chakravarthy R (2011). Comparison of the linear dimensional accuracy of denture bases cured the by conventional method and by the new press technique. Indian J Dent Res.

[22] Venus H, Boening K, Peroz I (2011). The effect of processing methods and acrylic resins on the accuracy of maxillary dentures and toothless denture bases: an in vitro study. Quintessence Int.

[23] Consani RL, Domitti SS, Rizzatti Barbosa CM, Consani S (2002). Effect of commercial acrylic resins on dimensional accuracy of the maxillary denture base. Braz Dent J.

[24] Sergio S, Robert E,  Ogle  Ogle (1999). Comparison of accuracy between compression and injection molded complete dentures. J Prosthet Dent.

[25] Alkhatib MB, Goodacre CJ (1990). Comparison of microwave-polymerized denture base resins. Int J Prosthodont.

[26] Lee CJ, Bok SB, Bae JY, Lee HH (2010). Comparative adaptation accuracy of acrylic denture bases evaluated by two different methods. Dent Mater J.

[27] Jagger DC, Harrison A, Jandt KD (1999). The reinforcement of dentures. J Oral Rehabil.

[28] Grajower R, Goultschin J (1984). The transverse strength of acrylic resin strips and of repaired acrylic samples. J Oral Rehabil.

[29] Jagger RG (1978). Effect of the curing cycle on some properties of a polymethylmethacrylate denture base material. J Oral Rehabil.

[30] Harrison A, Huggett R (1992). Effect of the curing cycle on residual monomer levels of acrylic resin denture base polymers. J Dent.

[31] Reis JM, Vergani CE, Pavarina AC, Giampaolo ET, Machado AL (2006). Effect of relining, water storage and cyclic loading on the flexural strength of a denture base acrylic resin. J Dent.

[32] Barbosa DB, de Souza RF, Pero AC, Marra J, Compagnoni MA (2007). Flexural strength of acrylic resins polymerized by different cycles. J Appl Oral Sci.

[33] Uzun G, Hersek N (2002). Comparison of the fracture resistance of six denture base acrylic resins. J Biomater Appl.

[34] Ganzarolli SM, de Mello JA, Shinkai RS, Del Bel Cury (2007). Internal adaptation and some physical properties of methacrylate-based denture base resins polymerized by different techniques. J Biomed Mater Res B Appl Biomater.

[35] Hamanaka I, Takahashi Y, Shimizu H (2011). Mechanical properties of injection-molded thermoplastic denture base resins. Acta Odontol Scand.

[36] Ucar Y, Akova T, Aysan I (2012). Mechanical properties of polyamide versus different PMMA denture base materials. J Prosthodont.

